# From the Editor’s desk

**DOI:** 10.4103/0970-2113.68302

**Published:** 2010

**Authors:** Virendra Singh

**Affiliations:** *Editor, Lung India, editor@lungindia.com*

Soon, I realized that editor of ‘Lung India’- the hallmark research journal, is a seat with more responsibility and challenges than laurels. To follow the benchmark set by the vanguard editors like Late Prof. C. V. Ramakrishnan, Prof. C. N. Deivanayagan, Prof. V. K. Vijayan, Prof. S. R. Kamat, Prof. P. S. Shankar, Prof. P. Ravindran and Prof. S. K. Jindal, who are luminaries and the vow to make the journal more useful to readers, is a tough challenge. But it can be accomplished with the combined vision and humble efforts of the editorial board.

We have thought to introduce the concept of section editor in the journal to harness the potential of entire board. It is very encouraging that we are receiving a number of innovative suggestions. Let us dedicate our wisdom and efforts, in a collective pack, to take ‘Lung India’ to new heights.

We have also planned to include color pictures to improve visibility of the articles from now onwards. This will be on shared basis. Fifty percent (INR 1000 for a colored page) of charges will be borne by the willing author.

Abstracts of Lung India articles published after 2004 are available on PubMed. We are trying to upload on PubMed, all the articles published in the Lung India since its inception. For this purpose hard copies of all previous issues of Lung India were provided with generous help from Prof. S. R. Kamat.

I solicit your thoughtful suggestions and the research articles which I am confident, would, for sure, help making the journal more purposeful.

